# Inorganic cesium lead mixed halide based perovskite solar materials modified with functional silver iodide

**DOI:** 10.1038/s41598-022-11729-0

**Published:** 2022-05-12

**Authors:** Vincent Obiozo Eze, Lucas Braga Carani, Haimanti Majumder, M. Jasim Uddin, Okenwa I. Okoli

**Affiliations:** 1 High-Performance Materials Institute, FAMU-FSU College of Engineering, 2525 Pottsdamer Street, Tallahassee, FL 32310 USA; 2 Photonics and Energy Research Lab, Department of Chemistry, University of Texas Rio Grande Valley, 1201 W University Drive, Edinburg, TX 78539 USA

**Keywords:** Electrocatalysis, Photocatalysis, Solar cells

## Abstract

Inorganic CsPbIBr_2_ perovskites have recently attracted enormous attention as a viable alternative material for optoelectronic applications due to their higher efficiency, thermal stability, suitable bandgap, and proper optical absorption. However, the CsPbIBr_2_ perovskite films fabricated using a one-step deposition technique is usually comprised of small grain size with a large number of grain boundaries and compositional defects. In this work, silver iodide (AgI) will be incorporated as an additive into the CsPbIBr_2_ perovskite precursor solution to prepare the unique perovskite CsI(PbBr_2_)_1-*x*_(AgI)_*x.*_ The AgI additive in the precursor solution works as a nucleation promoter which will help the perovskite to grow and merge into a continuous film with reduced defects. With detailed characterizations, we found that incorporating AgI additive resulted in a uniform perovskite film with fewer grain boundaries, increased grain size, crystallinity, optical absorption while decreasing carrier recombination and trap density. Using the AgI in an optimum amount, we fabricated CsPbIBr_2_ perovskite solar cells (PSCs) with a simple structure and achieved a power conversion efficiency (PCE) of 7.2% with a reduced hysteresis index. This work offers an alternative approach towards preparing high-quality CsPbIBr_2_ perovskite films for solar cells with higher stability and other optoelectronic applications.

## Introduction

Organic–inorganic metal halide perovskites are considered promising materials for potential applications in photovoltaics (PV), photodetectors, light-emitting diodes, and other optoelectronic applications^1–4^. The reason behind this vast applicability is their excellent optoelectronic properties, such as strong optical absorption, small exciton binding energy, large diffusion length, and ambipolar charge mobility^5–9^. The basic formula of organic–inorganic hybrid perovskite is ABX_3,_ where A and B are organic/inorganic cation, and X is an anion. Currently, the power conversion efficiency (PCE) of perovskite solar cells (PSCs) have soared up to 25.5%^10–13^, rivaling that of the commercial crystalline silicon, cadmium telluride (CdTe), and copper indium gallium selenide (CIGS) thin-film solar cells^14,15^. Despite the meteoric rise in the PCEs of the organic–inorganic-based PSCs, it is still dealing with the problem of poor thermal stability. Moreover, significant decomposition after annealing methylammonium lead halide (MAPbI_3_) perovskite at 85 °C resulting in phase separation of MAPbI_3_ into PbI_2_ and MAI has been widely reported^16–18^.

Recently, all inorganic cesium metal halide perovskites (CsPbI_*x*_Br_*3-x*_ (*x* = 0–3)) have attracted considerable attention as a rising light harvester due to their superior thermal stability over the widely used organic–inorganic metal halide perovskites, as well as their potential for use in PSCs and other optoelectronic devices^19–22^. However, the PCE of CsPbI_*x*_Br_3-*x*_ based PSCs is still not as high as the organic–inorganic lead halide perovskites. This can be attributed to poor film quality with defects and trap sites that resulted in a low short circuit current and poor phase stability^23,24^. Among the inorganic CsPbI_*x*_Br_3-*x*_ based perovskites, CsPbBr_3_ demonstrates superior stability under environmental stresses^25–27^ but has a large bandgap of about 2.3 eV, which is not ideal for solar cell applications. The CsPbI_3_, and CsPbI_2_Br exhibit comparatively narrow bandgaps of about 1.73 and 1.92 eV, respectively, but their thermal stability markedly deteriorates when they are exposed to high environmental stress like moisture, heat, ultraviolet radiation, etc. Interestingly, the CsPbIBr_2_ perovskite have balanced features, such as a bandgap of 2.05 eV and phase stability which makes it the most promising materials for optoelectronic devices. Although the poor film quality of the one-step solution-processed CsPbIBr_2_ perovskite hinders optimization of the device performance, additive engineering that involves the metal ion doping approach has been proven to be an effective strategy to improve the surface morphology, crystallinity, and photophysical properties and reduce the defects of CsPbI_*x*_Br_3-*x*_ perovskites^22,28^. This method is one of the most facile perovskite film preparation methods and plays a significant role in creating homogenous nucleation sites to enhance the crystallization rate, enlarging crystals to some extent, and modifying the surface energy to control the crystal growth directions of the perovskite^11,29–34^. To date, metal ions, such as Mn^2+^^30,35^, Bi^3+^^36^, Li^+^^37^, Sr^2+^^16^, Zn^2+^^38^, Eu^2+^^39^, Ni^2+^^11^ Ba^2+^^28^ have been incorporated into the perovskite crystal lattice to achieve improved films.

Zhao et al. used Sn^2+^ doping in CsPbIBr_2_ based PSCs and achieved a PCE of 11.33%^40^. The high PCE achieved by the Sn^2+^ doped PSCs was speculated to result from high short-circuit density (*J*_sc_) due to broadened light response range rather than decreased grain boundaries and compositional defect densities in the intrinsic CsPbIBr_2_ perovskite film^37^. In recent studies, it has been demonstrated that by using a preheating assisted spin-coating method, light processing, intermolecular exchange, interface passivation, and the combination of additive and anti-solvent dripping approach, the compositional defects of the CsPbIBr_2_ perovskite devices can be suppressed, and its PCE^15,25,41–43^ is increased. Although the above-mentioned methods are more efficient CsPbIBr_2_ PSCs, their approaches may be difficult to reproduce due to the multiple complex steps of perovskite film preparation. Therefore, it became essential to develop a simple and structured way to prepare uniform and high-quality CsPbIBr_2_ perovskite films that exhibit large grains, fewer grain boundaries, low defects density. Shahbazi et al. successfully modified the lattice structure to considerably improve the crystallinity, morphology, electronic properties of organic–inorganic hybrid perovskites (CH_3_NH_3_PbI_3_), as well as device performance using AgI additive^44^. However, the influence of AgI additive on CsPbI_*x*_Br_3-*x*_ perovskites for a photovoltaic application has not been investigated.

Herein, we prepared AgI modified CsPbIBr_2_ perovskite films using a simple, one-step solution process. The influence of AgI additive on the structural, morphological, optical, and electronic properties of the CsPbIBr_2_ perovskite film was systematically investigated by examining different characterizations. We observed that the AgI additive markedly improved the surface coverage, grain size, crystallinity, and photophysical properties of the perovskite film. The AgI additive has also improved charge extraction and reduced the defect density in the perovskite film, as well as improved the solar cell's performance. We fabricated PSCs using a ubiquitous structure to assess the effect of AgI additive on the device performance. The best-performing device achieved a PCE of 7.20% with a reduced hysteresis index. Our study provides a novel and facile strategy for morphology and crystallization controls and reduces defects in the CsPbIBr_2_ perovskite films for fabricating efficient PSCs and other optoelectronic devices.

## Materials and methods

### Materials and reagent

Fluorine-doped tin oxide (FTO) glass substrates (sheet resistance: 12–15 Ω/sq.) were purchased from MSE supplies. Liquid detergent (Hellemanex), Titanium (IV) Chloride (TiCl4, Alfa Aesar). All other chemicals were purchased from Sigma Aldrich and were used as received.

### Device fabrication

The patterned FTO-coated glass substrates were used, an electron transporting layer (ETL, compact TiO_2_) was prepared as reported elsewhere^45,46^. The CsPbIBr_2_ perovskite films were prepared by mixing CsI (1 M) and PbBr_2_ (1 M) in dimethyl sulfoxide (DMSO) (1 ml) in a nitrogen-filled glovebox and spin-coated on top of the compact TiO_2_/FTO substrates at 1500 rpm for 45s^22^. The perovskite films were prepared with AgI in mass percentages with respect to PbBr_2_ as the pristine perovskite^22,44^. The perovskite films were thermally annealed at 70 °C for 2 min, followed by 280 °C for 10 min. The hole transport layer (HTL, Spiro-OMeTAD) was prepared as reported elsewhere^45–49^. The contact electrode was deposited by thermal evaporation of Au metal with a thickness of 100 nm at 3.4 × 10^–4^ Pa. The solar cells' active areas were 0.06 cm^2^.

### Materials and device characterization

The morphology and particle size of the perovskite films were determined using scanning electron microscopy (SEM). SEM images were taken by a high-resolution field emission scanning electron microscope (FESEM) (JEOL 7401F). The crystalline structures of the as-prepared materials were characterized by powder X-ray diffraction (XRD) pattern (Scintag Pad-V, XRD powder diffractometer, graphite monochromatic Cu Kα radiation). The X-ray photoemission spectroscopy (XPS) was performed with an Mg Kα (1253.6 eV) X-ray source (Perkin Elmer). The absorption spectra of the films were measured using an ultraviolet–visible (UV–vis) spectrophotometer (Agilent Varian Cary 5000). The current density–voltage (*J*–*V)* measurements of the PSCs were recorded on a Keithley 2400 source measurement unit, IV5 solar cell I-V measurement system (PV Measurements, Inc.) under a simulated AM 1.5G illumination (Newport Oriel Instrument U.S.A, 94022A). Incident photon-to-electron conversion efficiency (IPCE) measurements were performed using light from the 300 W xenon lamp passed through a cornerstone 260 monochromator (Newport, 74,125) onto the cells, and the light scanned from 300 to 800 nm in 5 nm intervals. Incident light intensity and photocurrent were measured using a power meter (Newport, 2936-C) and Oriel 71580 Silicon Detector Head (Newport).

## Results and discussion

**Figure 1:**
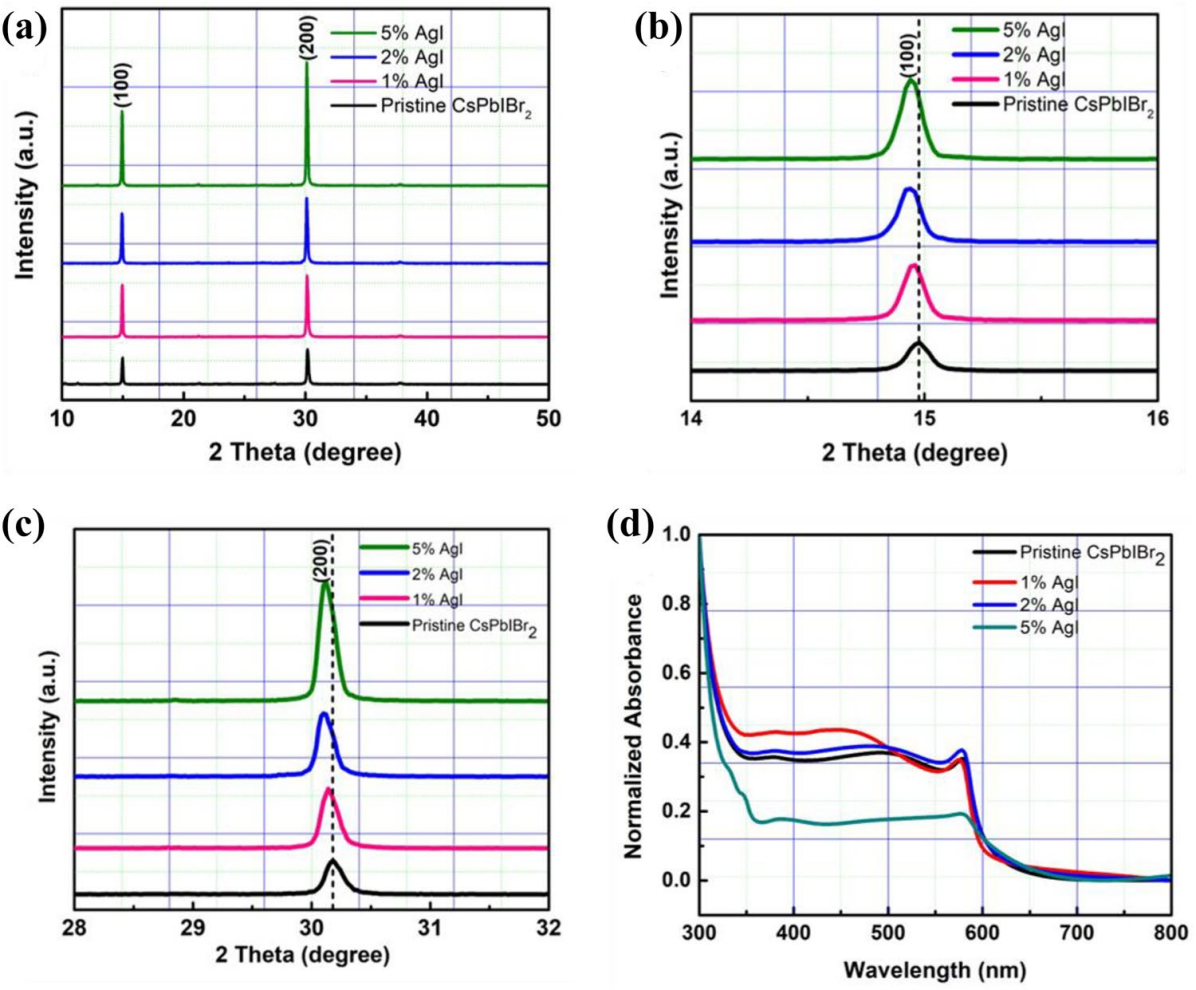
(**a**) XRD pattern of CsPbIBr_2_ perovskite films with and without AgI doping. (**b**) (100) peaks shift of the perovskite films. (**c**) (200) peaks shift of the perovskite films. (**d**) UV–vis spectra of with and without AgI doping.

**Figure 2:**
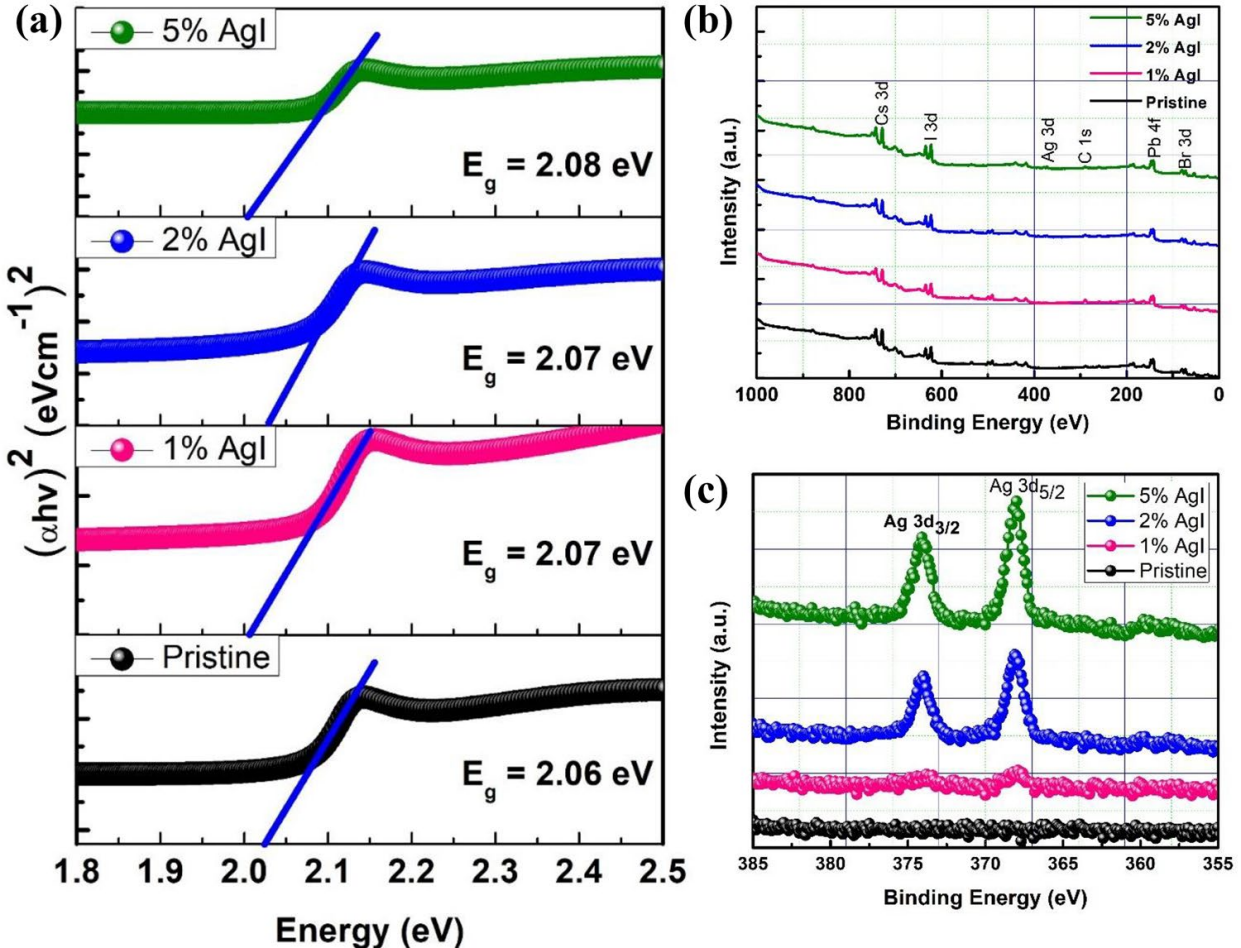
(**a**) Tauc plots of CsPbIBr_2_ perovskite films with and without AgI doping. XPS results for undoped and AgI doped CsPbIBr_2_ perovskite films. (**b**) Survey scan, (**c**) Ag 3d XPS spectra of undoped and AgI doped CsPbIBr_2_ perovskites.

The functional AgI nanoparticles was incorporated in different mass fractions (1.0, 2.0, 5.0%) into the precursor solution with respect to PbBr_2_ to prepare the CsI(PbBr_2_)_*1-x*_(AgI)_*x*_ perovskite films^22,44^. The photograph of the precursor solution with and without AgI additive is shown in Fig. S1. It was observed that the introduction of AgI led to the color change of the CsPbIBr_2_ perovskite precursor, suggesting the successful incorporation of the additive. For effective comparison, pure CsPbIBr_2_ perovskite films were also prepared. Both the CsPbIBr_2_ (without AgI doping) and the CsI(PbBr_2_)_*1-x*_(AgI)_*x*_ perovskite films were prepared by spin-coating with solutions containing cesium iodide (CsI) and lead bromide (PbBr_2_) in DMSO on top of a c-TiO_2_/FTO substrate. XRD characterization was performed to examine the influence of AgI on the crystallization of CsPbIBr_2_ perovskite films. Figure 1a shows the XRD pattern of the corresponding CsPbIBr_2_ perovskite films with different amounts of AgI. The main diffraction peaks around 14.93º and 30.16º can be assigned to the lattice plane of (100) and (200) of the α-phase perovskite^11,30^. The perovskite thin films showed no characteristic diffraction peaks of Ag in the bulk of the CsI(PbBr_2_)_*1-x*_(AgI)_*x*_ perovskite due to the smaller amount^22,50,51^. By comparison, the diffraction intensity of CsI(PbBr_2_)_*1-x*_(AgI)_*x*_ perovskite films increased and the full width at half maximum (fwhm) decreased with the increasing amount of AgI additive, indicating improved crystallinity of the perovskites^34,52^. We estimated the average crystallite sizes using the Scherrer's equation described below:

1 $$D=\frac{K\lambda }{\beta cos\theta }$$

where *D* is the average crystallite size (nm), λ is the wavelength (nm) of the X-rays, *β* is the fwhm (radian) of the diffraction peak, θ is the diffraction angle (degree), and *K* (0.94) is the shape factor. It can be observed that the crystallite sizes of the with AgI perovskite films are larger than the without AgI perovskite film, as shown in Fig. S2. This further confirms improved crystallinity for the AgI-doped perovskite films, which is advantageous in enhancing the optoelectronic properties of the perovskite films and device efficiency. The shifts in peak position were further investigated by analyzing the (100) and (200) peaks, as shown in Fig. 1b,c. The positions of the (100) and (200) XRD diffraction peaks shifted to a lower degree as the concentration of the AgI increased. This indicates an enlarged crystal lattice due to Ag doping while simultaneously showing that the Ag ions participate in the lattice formation^11,38^. Figure 1d shows the optical absorption spectra of the pristine CsPbIBr_2_ and AgI-doped CsPbIBr_2_ perovskite films. Note that conditions for all the fabricated films were the same except for the AgI doping concentration. Notably, an enhanced optical absorption was observed for the 1% and 2% AgI doped CsPbIBr_2_ perovskite films. The absorption seems to be reduced when the amount of AgI increases to 5%. Figure 2a shows the optical bandgap of the perovskite films calculated from the Tauc plot. The bandgap was increased for the AgI doped CsPbIBr_2_ perovskites, suggesting that the presence of the AgI dopants slightly modified the interactions with the ions in the network, leading to changes in the bandgap^53^. The XPS analysis was performed to identify the existence of AgI in CsPbIBr_2_ perovskite films. The survey scans for the binding energies from 0 to 1000 eV clearly showed the signals of Cs, Pb, I, Br, and C for both doped and undoped perovskite films (Fig. 2b). Figure 2c shows that Ag had been incorporated into the CsPbIBr_2_ perovskite. The peak intensities of Ag 3d_3/2_ and Ag 3d_5/2_ were observed to increase with the Ag content, which affirms the successful incorporation of AgI into the perovskite material^50,54^. Figure S3 shows the high-resolution spectra for Cs, Pb, I and Br. In comparison to the pristine sample, no apparent changes in the binding energy position for the AgI-doped CsPbIBr_2_ perovskite composition were observed, owing to the small substitution ratio of AgI^40^.

**Figure 3:**
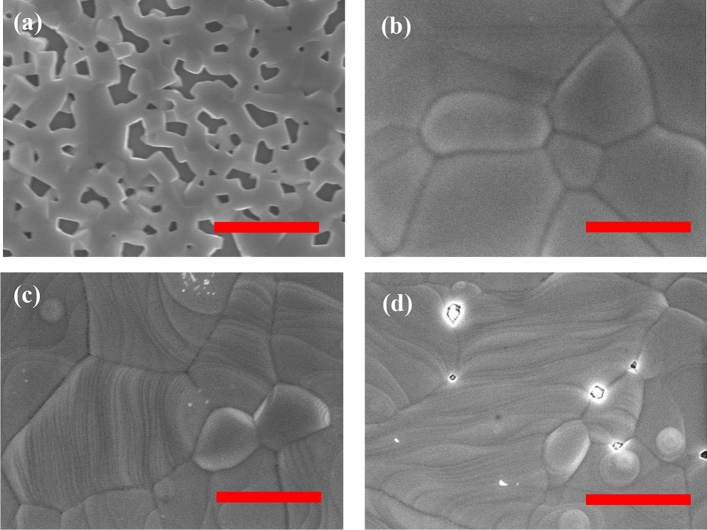
Low and high magnification SEM images of (**a**,**b**) pristine perovskite, (**c**,**d**) 1% AgI, (**e**,**f**) 2% AgI, (**g**,**h**) 5% AgI. Scale bars is 1 µm.

To examine the changes in the morphology of CsPbIBr_2_ perovskite films and the influence of Ag doping in controlling the crystal growth, we compared the SEM images of both doped and non-doped perovskite films. Figure 3a–d shows the SEM images of the pure and AgI based CsPbIBr_2_ perovskite films. Figure 3a shows that the CsPbIBr_2_ perovskite film exhibited a non-uniform morphology with voids on its surface. Previously reported work has suggested that the formation of such voids is probably due to the extremely slow crystallization of CsPbIBr_2_, where CsPbIBr_2_ species will crystallize from the precursor film containing plenty of DMSO molecules and then shrinks to leave behind isolated voids^22,25^. Interestingly, when 1% AgI is incorporated into the CsPbIBr_2_ perovskite, the morphology of the CsPbIBr_2_ perovskites showed marked changes.

**Figure 4:**
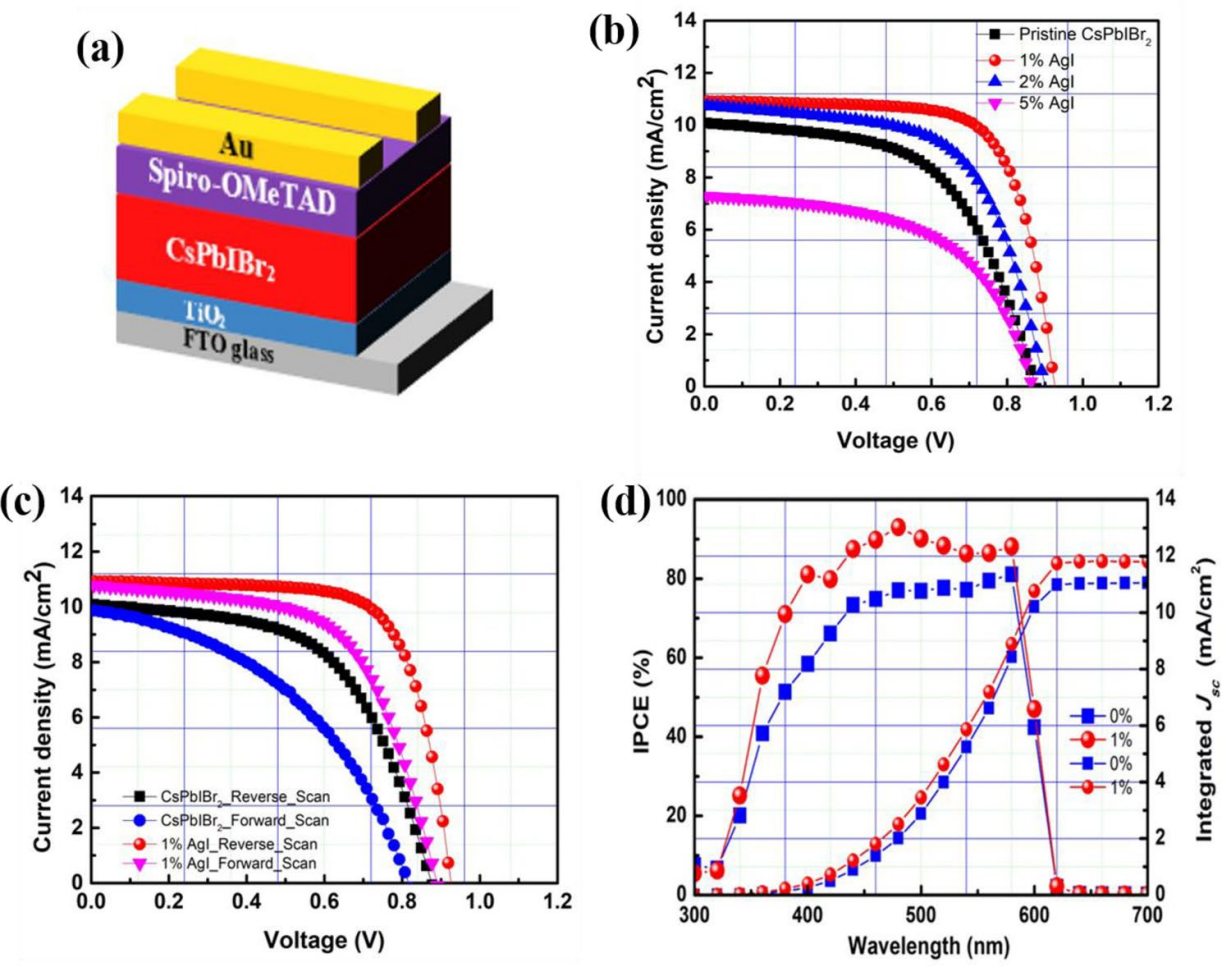
(**a**) Schematic diagram of the device architecture (**b**) Reserve scan of J-V curves for CsPbIBr_2_ perovskite PSCs with and without AgI incorporation measured under simulated AM 1.5 G illumination (**c**) Hysteresis behavior of pristine and 1% AgI CsPbIBr_2_ PSCs. (**d**) IPCE of the devices.

Large, uniform grains developing in marked pin-hole free films were observed in Fig. 3b. The 1% AgI perovskite film exhibited a compact and uniform morphology with larger grain size and fewer grain boundaries. These properties are expected to decrease the crystal defects and trap density while also reducing charge recombination and enhancing charge transport in the device^22,45–47,55–57^. Upon increasing the amount of AgI to 2% (Fig. 3c), the grain size was further enlarged, with the largest grains resulting from the 5% AgI dopant (Fig. 3d). However, we also observed that 2% and 5% AgI perovskite films showed wrinkles and wave-like features, which can be attributed to defects caused by excess AgI in the perovskite host^52^. Recently, we reported the possible mechanism for improving surface coverage for the CsI(PbBr_2_)_1-x_(AgI)_x_ perovskite films, as shown in Fig. 2^22^. Ag islands were formed for the precursor solution containing AgI additive upon depositing it on a substrate. The Ag seeds could act as crystal growth promoters for the perovskite to grow and merge into a continuous film with fewer grain boundaries and enhanced crystallinity^22,52,54,58^. To examine the impact of AgI doping on the device performance, a simple and ubiquitous planar PSCs based on FTO/c-TiO_2_/CsI(PbBr_2_)_1-*x*_(AgI)_*x*_/Spiro-OMeTAD/Au geometry was fabricated as presented in Fig. 4a. Table 1 summarizes the PV parameters of PSCs based on CsPbIBr_2_ perovskite films with and without AgI doping. Figure 4b shows the *J-V* characteristics of the AgI additive modified CsPbIBr_2_ and the pristine PSCs. The pristine device delivered a PCE of 5.2% (average value 4.50%) with a *J*_sc_ of 10.29 mA/cm^2^, a *V*_oc_ of 0.88 V, and *FF* of 0.57. The reason behind the lower efficiency of the pristine CsPbIBr_2_ PSC is poor perovskite film quality (see Fig. 4a). Moreover, insufficient coverage could facilitate a high frequency of shunt paths and allow contact between the electron transport layer (ETL) and hole transporting layer (HTL), thereby decreasing the PV parameters of the PSCs^48,56,59,60^. The 1% AgI doped device yielded the highest PCE of 7.2% (average value 6.85%) with a *J*_sc_ of 11.00 mA/cm^2^, a *V*_oc_ of 0.92 V, and a FF of 0.71, which are higher than the pristine device. It was noticed that with an increasing amount of the concentration of AgI doping beyond 2% led to a decrease in the PV performance. The higher PCE achieved by the 1% AgI doped device demonstrates that Ag doping plays an important role in efficiency enhancement due to the improved surface morphology, crystallinity, enlarged grain size, and fewer grain boundaries and defects. Figure 4c shows the forward scan (from a short circuit to an open circuit) and reverse scan (from an open circuit to a short circuit) for the pristine and 1% AgI doped CsPbIBr_2_ PSCs. Anomalous hysteresis of *J-V* measurements has been widely reported as one of the most critical issues in PSCs, which often gives rise to the overestimation of the solar's PCE. Typically, the origin of hysteresis can be from the charge-selective layers, trapping and de-trapping of charge carriers, ionic movement, or ferroelectric properties of the perovskite materials^47–49,61–65^. It is apparent that both devices showed hysteresis in *J-V* curves scanned in the reverse and forward direction. According to our experimental results, the additive AgI does not alleviate the hysteresis behavior in the PSCs; however, the hysteresis index (A) calculated using the equation below^66^ decreased from 0.34 (pristine CsPbIBr_2_ device) to 0.21 (1% AgI CsPbIBr_2_ device).

**Table 1:** Summary of PV parameters of the best performing devices together with the average PCE of the pristine and AgI based CsPbIBr_2_ PSCs measured under standard AM 1.5G illumination with a light intensity of 100 mW/cm^2^.

Device	*J*_sc_ (mA/cm^2^)	Voc (V)	FF	PCE (%)	Average PCE (%)
Pristine	10.29	0.88	0.57	5.2	4.50 ± 0.4
1% AgI	11.00	0.92	0.71	7.2	6.85 ± 0.2
2% AgI	10.94	0.90	0.61	6.0	5.72 ± 0.2
5% AgI	7.39	0.86	0.58	3.7	1.16 ± 0.7

2 $$A=\frac{PCE \left(reverse\, scan\right)-PCE(forward\, scan)}{PCE (reverse\, scan)}$$

The 1% AgI CsPbIBr_2_ PSC can have improved morphology with fewer grain boundaries with decreasing value of A, which can facilitate efficient charge carriers transport in the solar cell. Figure 4d shows the corresponding IPCE for the pristine and 1% AgI doped CsPbIBr_2_ PSCs. The 1% AgI device exhibited higher IPCE over the entire wavelength range than the controlled device, which further corroborates the UV–vis results. The enhanced IPCE for the 1% AgI doped device can be attributed to compact and large grains, which consequently also enhanced charge extraction and suppressed charge recombination in the device.

**Figure 5:**
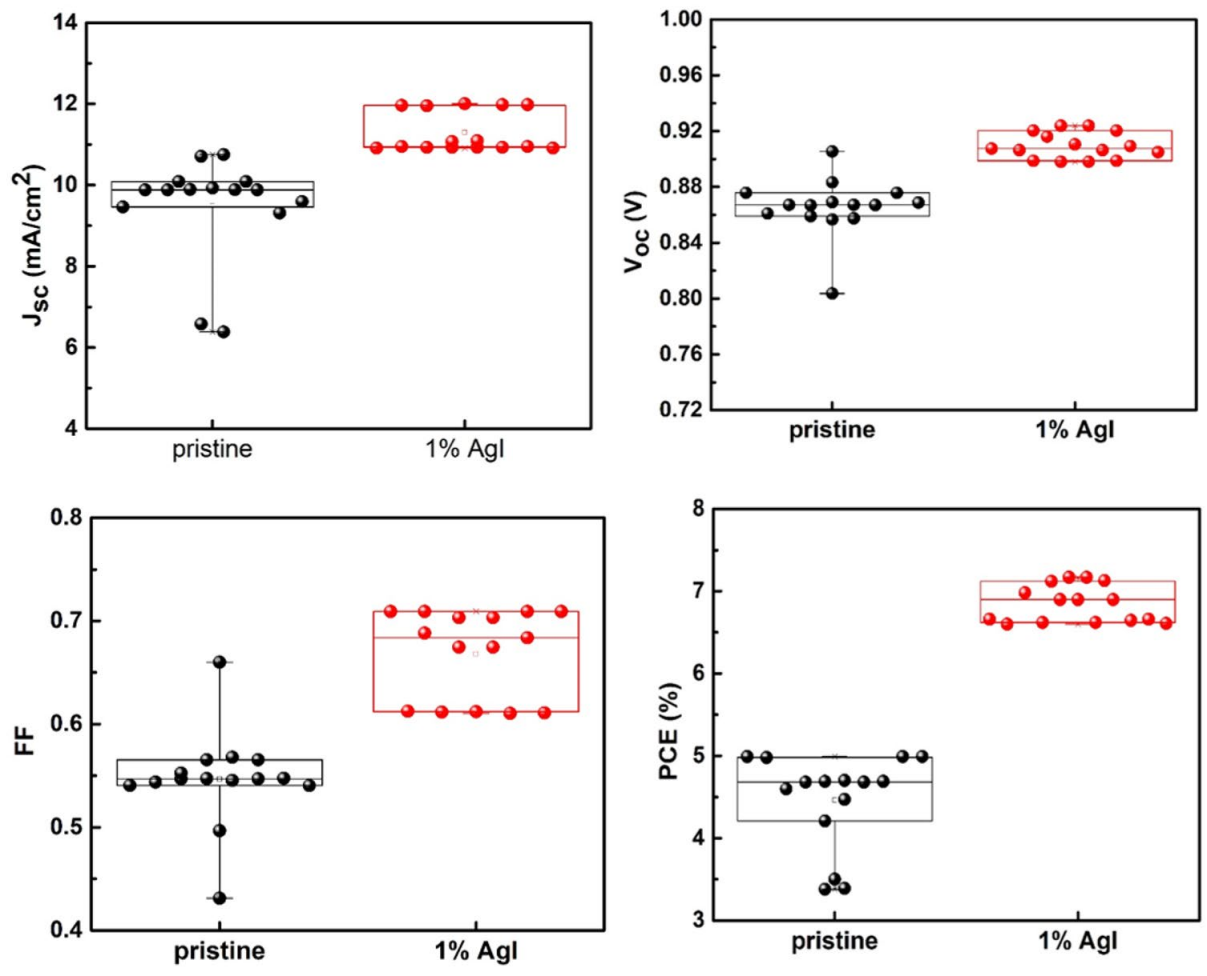
Statistical distribution of PV parameters for the pristine and AgI PSCs, based on 15 cells.

The calculated *J*sc values are 11.06 mA/cm^2^ and 11.83 mA/cm^2^ for the pristine and 1% AgI-doped perovskite devices, respectively, match well with the experimental *J*sc values provided in Table 1. The slight variation may stem from the spectral mismatch between different solar simulators^67,68^. The box plot of 15 cells for the pristine and 1% AgI doped CsPbIBr_2_ PSCs that present the statistical features of *J*_sc_, *V*oc, *FF*, and PCE is shown in Fig. 5. The box plot indicates the enhancement of performance more. The average PV parameters of 1% AgI doped cells are higher than the control devices, and further confirms that Ag doping is beneficial in enhancing the PV parameters of CsPbIBr_2_ PSC.

**Figure 6:**
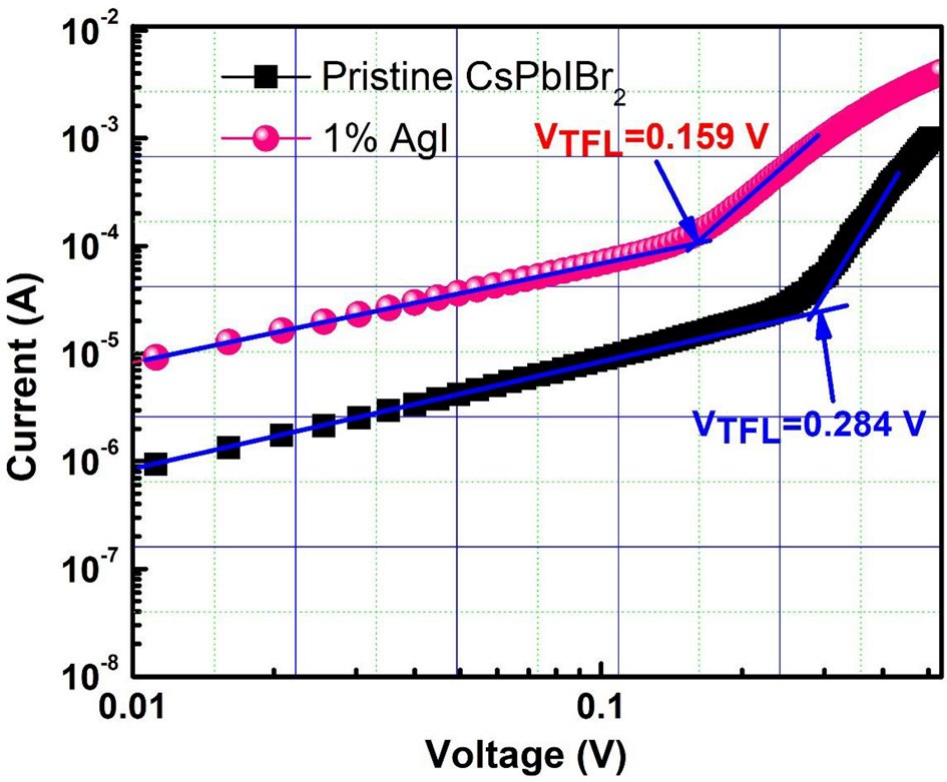
Hole-only devices films (FTO/PEDOT: PSS/CsPbIBr_2_/P3HT/Ag) for pristine, and 1% AgI CsPbIBr_2_ perovskite films.

Hole-only devices with a geometry of FTO/PEDOT:PSS/CsPbIBr_2_/P3HT/Ag, respectively, were fabricated in order to determine the trap state density (*n*_trap_). Figure 6 shows the plots of *I–V* curves under dark conditions. The trap state density can be determined from the trap-filled limit voltage (*V*_TFL_), according to the equation below^41,69^.

3 $${n}_{trap}=\frac{2\varepsilon {\varepsilon }_{0}}{e{d}^{2}}{V}_{TFL}$$

where ε is the relative dielectric constant of CsPbIBr_2_, which is approximately equal to 8^22,70^, *ε*_*0*_ is the constant of vacuum permittivity in free space*,* d is the thickness of the perovskite film and e is the electron charge*. V*_TFL_ is estimated from the *I–V* curves. Table S1 shows the *V*_TFL_ and *n*_trap_ values of pristine and 1% AgI doped CsPbIBr_2_ PSCs. The approximate *V*_TFL_ values for the hole only devices for the pristine and 1% AgI doped perovskite films were 0.159 V and 0.284 V, respectively. The *n*_trap_ for the hole-only devices for the pristine and 1% AgI doped CsPbIBr_2_ perovskite films were calculated to be 4.02 × 10^15^ cm^−3^ and 2.25 × 10^15^ cm^−3^, respectively (see Table S1). The defect densities for 1% AgI doped CsPbIBr_2_ perovskite devices were reduced in comparison to the pristine devices. The decrease in defect density for the 1% AgI doped device is attributed to the high-quality perovskite film with enhanced crystallinity, enlarging grains, and fewer grain boundaries.

## Conclusion

In summary, this paper reports improvement in the quality of CsPbIBr_2_ perovskite films by incorporating AgI additive as an effective strategy. We studied the effects of AgI additive on the morphology, crystallinity, optical properties, and defect density of the CsPbIBr_2_ perovskite films. Our investigation suggested that using the AgI additive in CsPbIBr_2_ perovskite film has improved the structural, morphological, and optoelectronic properties of the perovskite films. The introduction of AgI in the CsPbIBr_2_ precursor led to a uniform surface coverage of CsPbIBr_2_ perovskite film that exhibited larger grain size, improved crystallinity, and decreased defect densities and carrier recombination. To confirm the effectiveness and effect of the AgI modified CsPbIBr_2_ perovskite film on improving the PV properties of the solar cells. A simple planar structure was fabricated, and the 1% AgI device achieved a PCE of 7.20%, which is higher than the pristine device (5.2%). This work provides a useful strategy toward enhancing the film quality and optoelectronic properties of CsPbIBr_2_ perovskites for the fabrication of efficient PSCs and other optoelectronic devices.

## Supplementary Information

Supplementary Information.
