# Supplementary material for: Inorganic cesium lead mixed halide based perovskite solar materials modified with functional silver iodide
Source: Sci Rep. 2022 May 12;12:7794. doi: 10.1038/s41598-022-11729-0 (PMC9098910; doi:10.1038/s41598-022-11729-0)
Supplement: Supplementary file 1 — Supplementary Information. [file 41598_2022_11729_MOESM1_ESM.docx]

**Supporting Information**

**Inorganic Cesium Lead Mixed Halide Based Perovskite Solar Materials Modified with Functional Silver Iodide**

Vincent Obiozo Eze^1^, Lucas Braga Carani^1^, Haimanti Majumder^2^, M. Jasim Uddin^2^, Okenwa I. Okoli^1^*

^1^High-Performance Materials Institute, FAMU-FSU College of Engineering, 2525 Pottsdamer Street, Tallahassee, FL 32310, United States.

^2^Photonics and Energy Research Lab, Department of Chemistry, University of Texas Rio Grande Valley, 1201 W University Drive, Edinburg, Texas 78539, United States.

**Corresponding Author email:** **mohammed.uddin@utrgv.edu*,** [**okoli@eng.famu.fsu.edu**](mailto:okoli@eng.famu.fsu.edu)*****

FWHMs and average crystallite sizes of (100) peaks, (b) FWHMs and average crystallite sizes of the (200) peaks. The average crystallite sizes of the corresponding perovskites were estimated using Scherrer’s equation, High resolution XPS spectra for Cs, Pb, I and Br, Summary of Trap filled voltage and trap density values of pristine and 1% AgI CsPbIBr_2_ perovskite films.


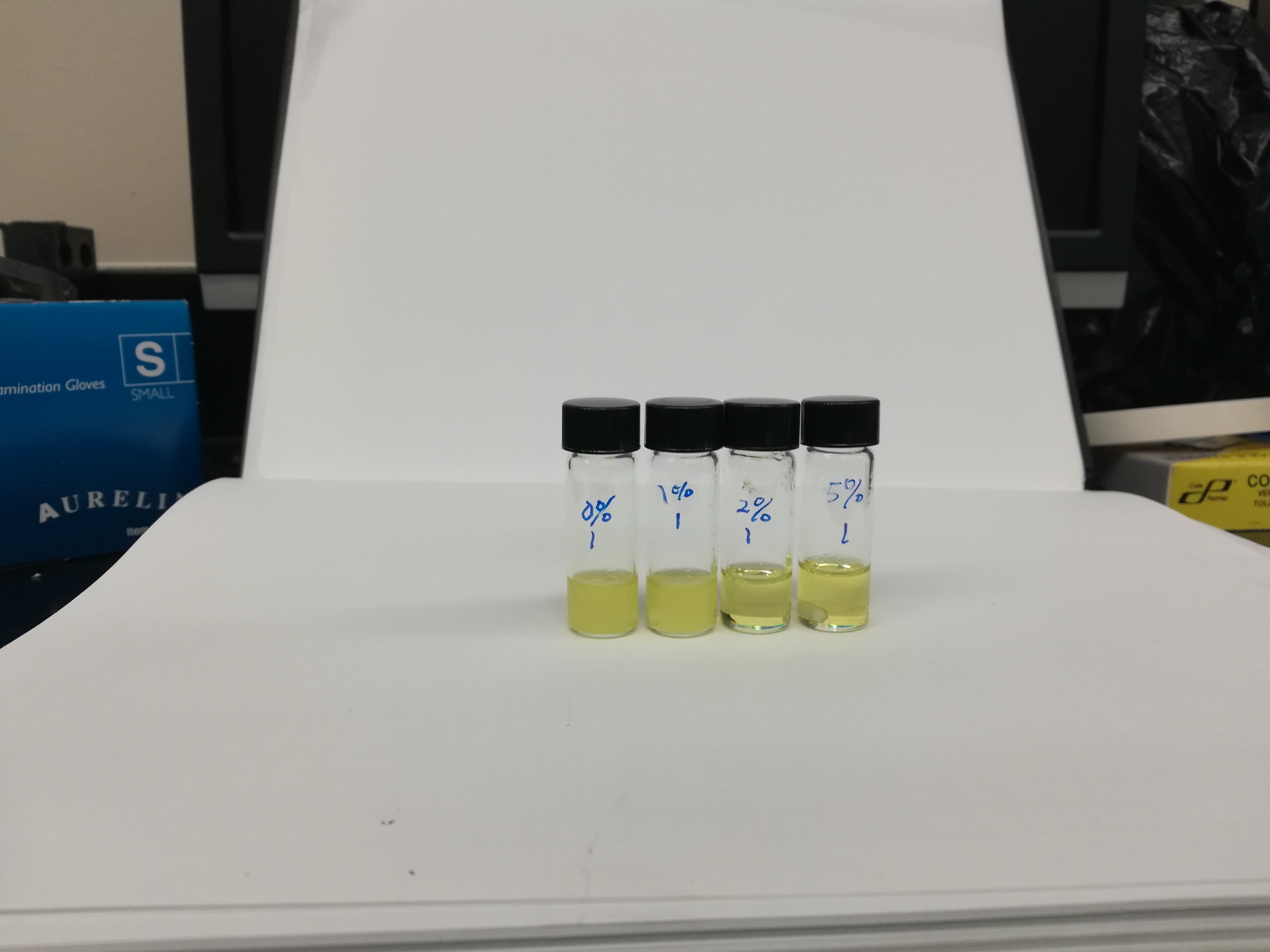


**Figure S1.** Precursor solution of with and without AgI additive incorpotation

**Figure S2.** (a) FWHMs and average crystallite sizes of (100) peaks, (b) FWHMs and average crystallite sizes of the (200) peaks. The average crystallite sizes of the corresponding perovskites were estimated using Scherrer’s equation.


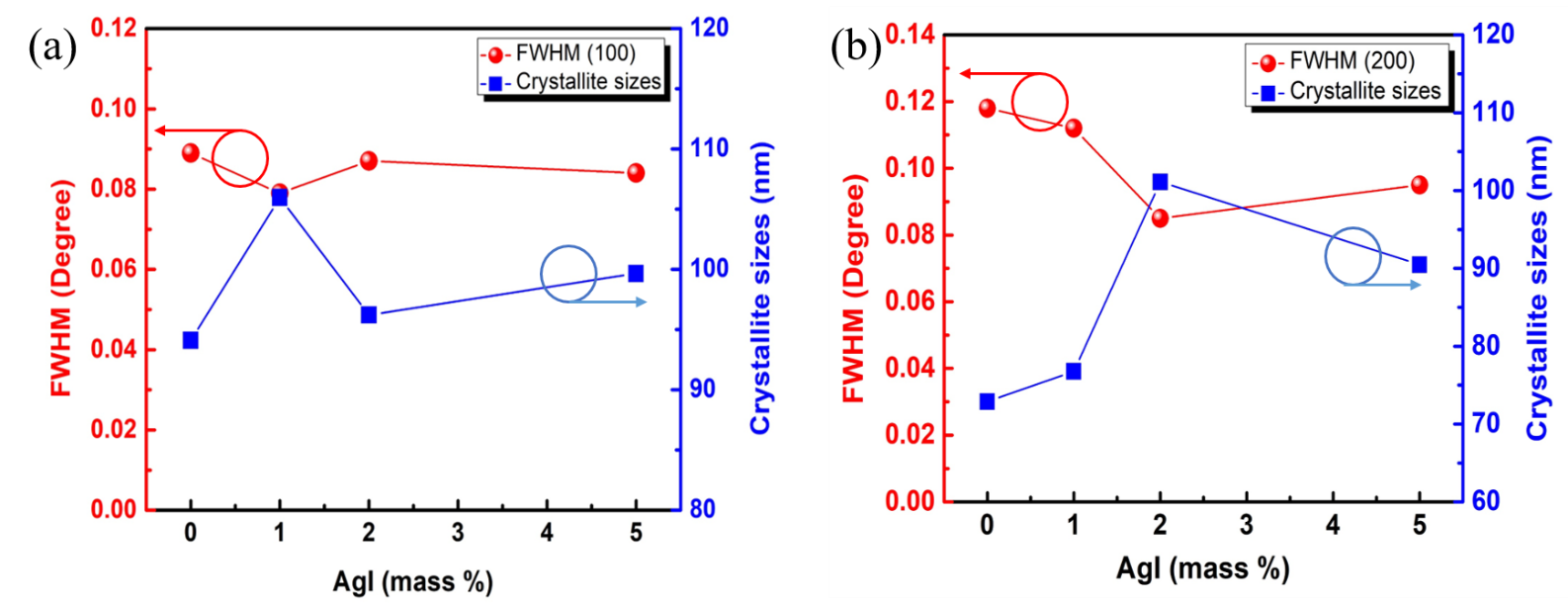


**Figure S3.** High resolution XPS spectra for Cs, Pb, I and Br.


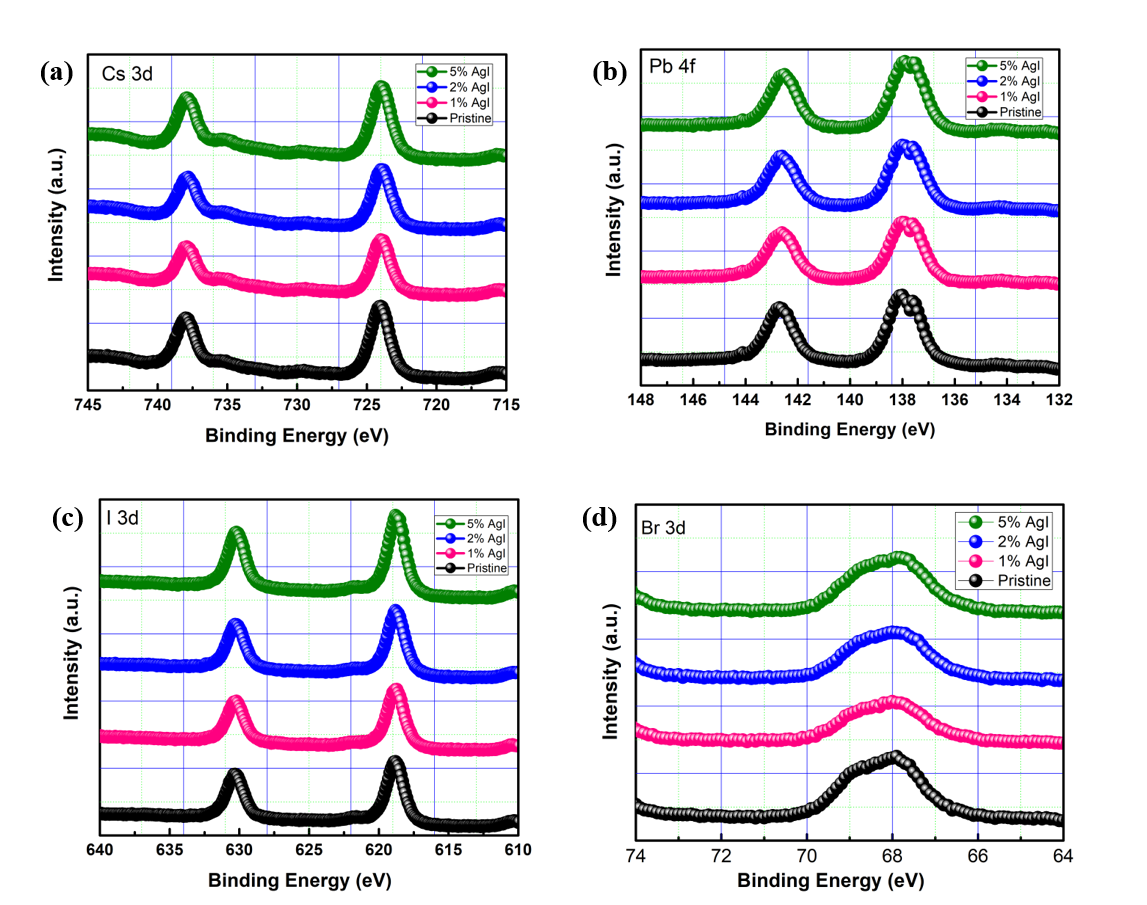


**Table S1.** Trap filled voltage and trap density values of pristine and 1% AgI CsPbIBr_2_ perovskite films

|  | Samples | V_TFL_ (V) | *n*_trap_ (cm^-3^) |
| --- | --- | --- | --- |
| Hole only devices | 0% | 0.284 | 4.02 × 10^15^ |
|  | 1% | 0.159 | 2.25 × 10^15^ |

*For the hole only devices, the configuration is FTO/PEDOT: PSS/perovskite/P3HT/Ag.
